# Renal Arterial Anatomy: Implications for Normothermic Machine Perfusion in Renal Transplantation

**DOI:** 10.1002/ca.24286

**Published:** 2025-05-07

**Authors:** Lily Mae Miller, Peter Douglas, Julie Glen, Emma Aitken

**Affiliations:** ^1^ College of Medical, Veterinary and Life Sciences University of Glasgow Glasgow UK; ^2^ Department of Radiology Queen Elizabeth University Hospital Glasgow UK; ^3^ Department of Renal Transplantation Queen Elizabeth University Hospital Glasgow UK

**Keywords:** donor, ex vivo normothermic perfusion, kidney, magnetic resonance angiography, normothermic machine perfusion, renal, renal anatomy, renal arteries, transplant

## Abstract

Normothermic machine perfusion (NMP) is a novel technology that has shown potential in viability assessment and reconditioning of donor organs. Normothermic machine perfusion is technically more challenging in kidneys with multiple renal arteries (RAs). This study aimed to characterize the anatomical variation in RAs with a view to the development of optimal equipment for RA cannulation in NMP. Magnetic resonance angiograms obtained from all potential living donors at our centre between 2018 and 2022 were evaluated with PACS 3D with vessel analysis. Three independent reviewers measured RA characteristics (number, cross‐sectional area, diameter, distance, angulation). A total of 618 kidneys were included for analysis. Kidneys were categorized into five groups based on the anatomical configuration of arteries. 75.4% had a single RA (group one). One quarter of kidneys were found to have multiple RAs, with inferior accessory arteries the commonest variation. Of the 139 kidneys with multiple RAs (24.6%): 5.3% had two equal sized RAs (group two), 5.5% had a superior accessory artery (group three), 11.7% an inferior accessory artery (group four), and 2.1% had three RAs (group five). Left RAs were of larger diameter, but right RAs were longer (*p* < 0.001). The number of arteries supplying the right kidney significantly predicted the number of arteries supplying the left kidney (*β* = 0.15). 23.7% of kidneys with two RAs and 30.8% with three RAs had distances between vessels > 28 mm (length of the existing clamp used for perfusion). 19.1% of main RAs had originated from the aorta at angles ≥ 135°. These findings highlight the insufficiencies with existing NMP equipment for cannulation of the RA. A larger clamp may facilitate perfusion of more kidneys with multiple vessels, whilst soft, flexible cannulae are likely to be needed to accommodate the wide range in angulation of RA origin demonstrated.

## 1 Introduction

Transplantation is the optimal treatment for end‐stage renal failure. It provides lower morbidity and mortality and improved quality of life compared with alternative forms of renal replacement therapy, such as hemodialysis (Tonelli et al. 2011; Chaudhry et al. 2022). Around 3000 adult kidney‐only transplants are performed in the UK each year (NHS Blood and Transplant Service 2022a). However, a shortage of donor organs limits deceased donor transplantation, resulting in prolonged patient waiting times and an expanding transplant waiting list. The median waiting time for a deceased donor kidney transplant in the UK between 2015 and 2019 was 550 days (NHS Blood and Transplant Service 2022b). As of March 2024, a total of 6250 patients across the UK are active on the kidney transplant waiting list (NHS Blood and Transplant Service 2022b). During the 2021/22 period, over 260 patients died whilst on this list, many likely related to progression or complications of renal disease (NHS Blood and Transplant Service 2022b). Kidneys from extended criteria donors (ECD) and donation after circulatory death (DCD) donors are being increasingly utilized to expand the donor pool and overcome the problem of supply and demand imbalance. These organs are perceived to be less favorable; predicted to be more susceptible to negative effects of ischaemic reperfusion injury (IRI); associated with increased rates of delayed graft function (DGF) and primary non‐function (PNF) and overall confer less successful long‐term transplant outcomes (Hosgood et al. 2021, 2017, 2013; Hamelink et al. 2022; Elliott et al. 2021; Pool et al. 2021; Kaths et al. 2017; Nicholson and Hosgood 2013). This heightened potential for undesirable outcomes from ‘marginal’ grafts has prompted identification of better ways to assess kidneys in regard to viability and explore potential preservation/reconditioning strategies prior to transplantation, negating the impact of IRI. Increased surgeon confidence in the viability of organs pre‐transplant may increase the number of available donor kidneys through utilization of kidneys which may have previously been discarded, presumed to be suboptimal (Hamelink et al. 2022; Elliott et al. 2021). Emerging novel kidney perfusion techniques may facilitate this assessment.

**FIGURE 1:**
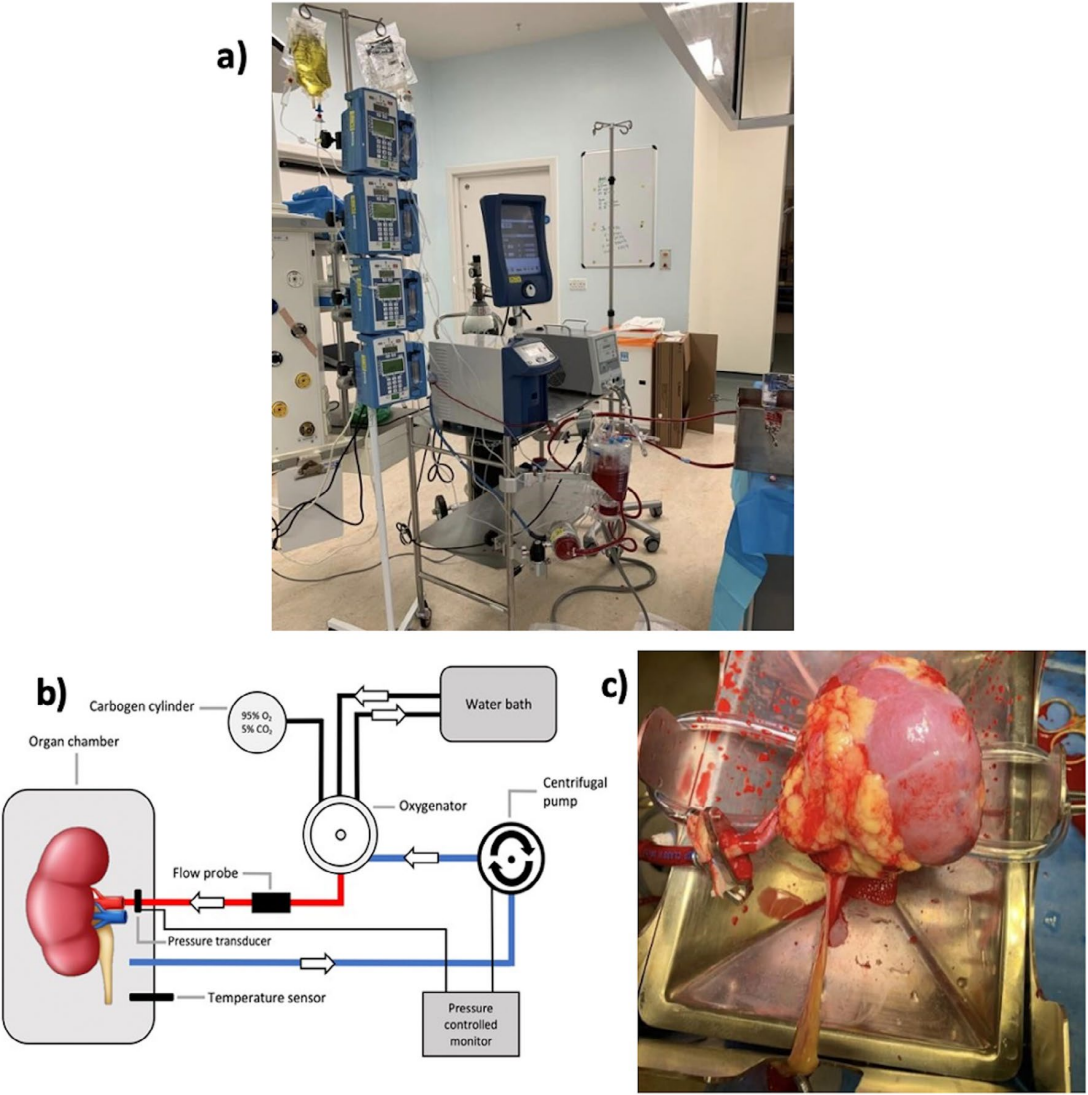
Normothermic machine perfusion (NMP). (a) The NMP circuit in use at our institution. On the left are infusion pumps permitting infusion of nutrients and drugs to maintain acid–base balance. The centre of thee image shows the main NMP circuit connected via plastic tubing to the kidney in a specially designed metal chamber on the right. (b) Schematic diagram of the NMP circuit demonstrating the oxygenator and centrifugal pump. The circuit is pressure, flow and temperature controlled. Credit to M. Pool and T. Hamelink et al. and permission from the Public Library of Science (PLOS) journal for reuse (Pool et al. 2021). (c) Deceased donor kidney (deceased) being perfused on the NMP circuit at our centre. On the left of the picture, the Walters clamp is visible holding the aortic patch from which a single main renal artery originates. This is a healthy, well‐perfused deceased donor kidney, demonstrated by the pinkish color and low resistance.

One such novel viability assessment technique is Normothermic Machine Perfusion (NMP) (also known as Ex Vivo Normothermic Perfusion (EVNP)). Circulation of a warm, oxygenated, nutrient‐enriched perfusion solution throughout the renal vasculature, as outlined in Figure 1, provides a pseudo‐physiological organ preservation environment. Replenishment of ATP stores within the kidney restores normal cellular metabolism, negating the hypoxic damage induced by cold ischaemia and static cold storage (Elliott et al. 2021). This technology may have the potential to reduce rates of DGF, and its benefits in organ viability assessment, protective effects, and reconditioning the kidney prior to reimplantation are being investigated (Hosgood et al. 2021, 2017, 2013, 2023; Hamelink et al. 2022; Elliott et al. 2021; Pool et al. 2021; Kaths et al. 2017; Nicholson and Hosgood 2013; Hosgood and Nicholson 2011, 2024).

Results of the first in‐man renal transplantation following NMP were reported by Nicholson and colleagues in 2011 (Hosgood and Nicholson 2011). Both kidneys from the same donor were transplanted into two different recipients. One kidney received NMP, and its paired counterpart received traditional static cold storage (SCS) preservation on ice. Although both transplants were ultimately successful, the normothermically perfused kidney was associated with improved early transplant function with less acute tubular necrosis and better initial urine production (Hosgood and Nicholson 2011). Subsequently, Hosgood et al. published a pilot study of 18 kidneys from ECDs perfused on NMP, demonstrating both the safety and feasibility of NMP in renal transplantation. They also demonstrated a significantly lower rate of DGF in ECD kidneys in the NMP group compared to the control group (5.6% vs. 36.2%) (Nicholson and Hosgood 2013). A larger randomized controlled trial (RCT) was published in 2023 comparing outcomes of 338 DCD kidneys treated with 1 h of NMP after SCS compared with SCS alone. In this study, no difference in the rate of DGF was observed (Hosgood et al. 2023). Nevertheless, NMP continues to provide exciting opportunities for viability assessment and organ reconditioning.

Much work is being undertaken to determine the perfect NMP regime in terms of timing and perfusion composition; however, the NMP circuit itself remains crude, and we continue to look at ways to improve it. Successful and timely cannulation of the renal artery (RA) without causing injury remains a major challenge. This is a particular problem when multiple RAs or complex renal anatomy exist. For example, multiple RAs or RAs with very early bifurcation introduce challenges with fixing a cannula suitable for perfusing both vessels. Variation in expertise and confidence associated with handling multiple RAs leads to subjectivity in decision making. Within the recent RCT by Hosgood et al., the decision whether or not to recruit kidneys with multiple vessels was left at the discretion of the local investigator (Nicholson and Hosgood 2013; Hosgood et al. 2023). Within this trial, 8.2% (*n* = 14) of kidneys in the NMP arm did not receive NMP due to the inability to secure a cannula into or around the RA (Hosgood et al. 2023).

Classically, kidneys are described as being supplied by a single RA originating from the abdominal aorta between the levels L1 and L2. However, in reality, we know that this is not the case, with several studies reporting the presence of multiple RAs. Most authors report 20%–30% occurrence rates (Pradhay et al. 2021; Sampaio and Passos 1992).

The aim of this study is to characterize the anatomy of RAs within a contemporaneous cohort, taking into account specific anatomical features (e.g., rate of occurrence of multiple RAs; distance between origins of multiple vessels; angulation of RA at its origin from the aorta etc.) that might have relevance to cannulation for NMP. It is hoped that results from this study will aid the development of tools and equipment to facilitate NMP in kidneys with complex arterial anatomy, thereby expanding the pool of organs suitable for viability assessment/reconditioning techniques via NMP. Ultimately, it is hoped that this could increase transplant numbers, improve transplant outcomes, and reduce the number of patients dying while on the transplant waiting list.

## 2 Materials and Methods

### 2.1 Aim

To describe and characterize variations in renal arterial anatomy as it might relate to successful cannulation for NMP.

### 2.2 Patients

The living kidney donor population was assumed to be representative of the wider Scottish donor population for renal anatomy. We identified all potential living kidney donors in the West of Scotland who reached the imaging stage in the work‐up pathway for living donation over a 5‐year period (2018–2022) from a database prospectively maintained by the local live donor transplant coordinators.

Contrast‐enhanced magnetic resonance angiography (CE‐MRA) is the imaging modality of choice for anatomical evaluation of living kidney donors in the West of Scotland. Patients were subsequently excluded if they did not receive CE‐MRA for clinical reasons, e.g., claustrophobia (these patients received computed tomography angiography (CTA) instead) or if CE‐MRA was of poor quality.

### 2.3 Imaging Analysis

**FIGURE 2:**
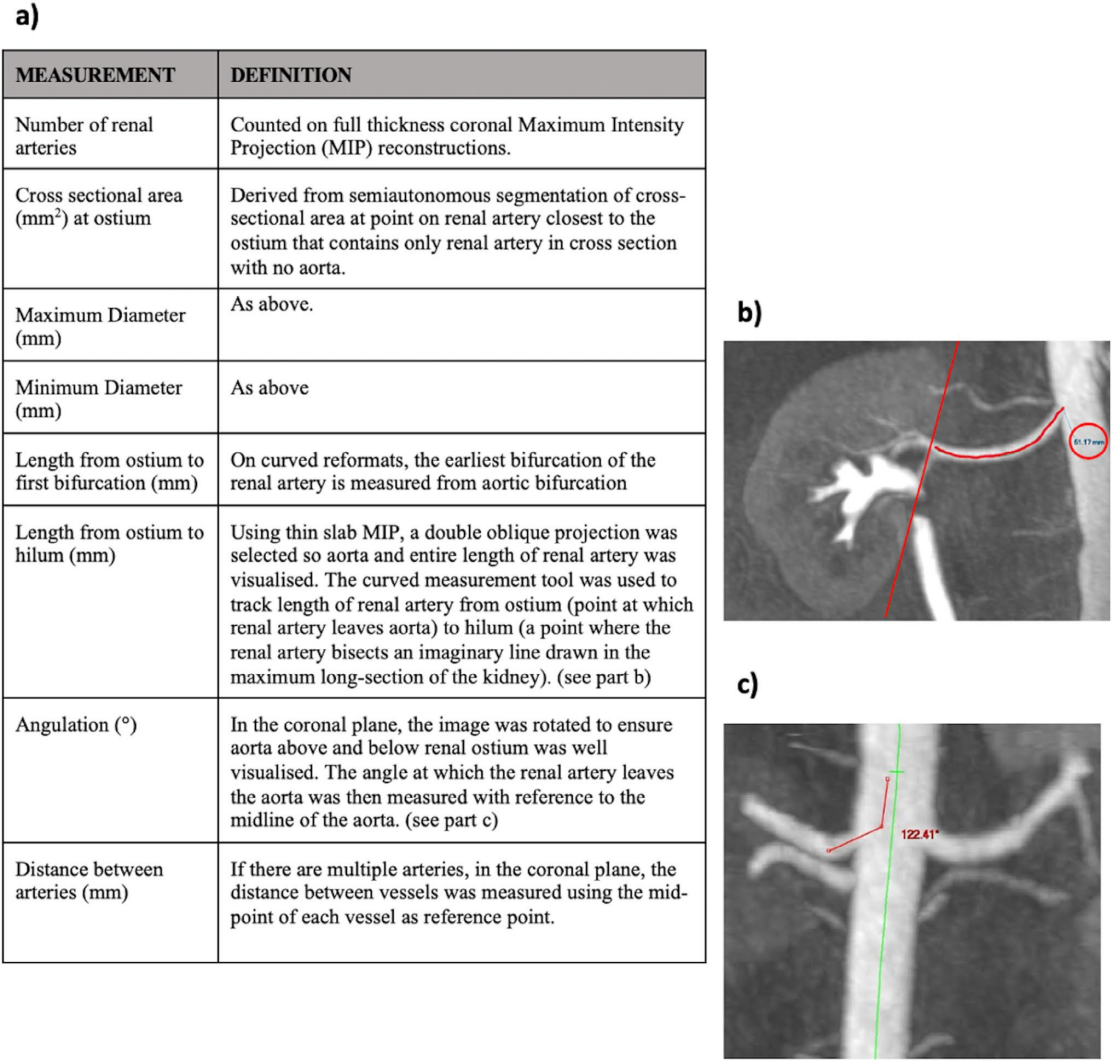
Measurement methods. (a) Definitions of arterial characteristics analyzed and the protocol of analysis used on VUE PACS. The standard operating procedure (SOP) can be found in Appendix A. (b) Example of procedure for measuring the length of a renal artery from the ostium to the hilum on CE‐MRA. The red line depicts how the main RA was traced using VUE PACS. (c) Example of procedure for measuring angulation on CE‐MRA. The green line depicts the centre of the aorta. The red line depicts how angulation was measured using VUE PACS utilizing this “centre line” as a reference point.

We obtained all CE‐MRA images according to the standard protocol for living donor work‐up: ‘Coronal and Transverse T2w TSE CE‐MRA of the abdominal aorta and renal arteries’. Contemporaneous reporting by the clinical radiology service at our centre informed the clinical decision about suitability to donate which was made independently of this study. Images were subsequently analyzed by a single investigator using the Vue‐PACS 3D and Vessel Analysis package (Version 12.2.2.1025). Data were collected on number of RAs, length, maximum and minimum diameters, cross‐sectional area at the hilum, and angulation as renal vessels came off the aorta. In cases of multiple vessels, distance between vessels was also recorded. We regarded each kidney as a separate entity (i.e., two kidneys per patient). Measurement methods and definitions of the arterial characteristics analyzed are detailed in Figure 2. Full details of the protocol for obtaining measurements are outlined in Appendix A.

We classified kidneys into five groups based on their anatomical configuration (single RA, two equal sized arteries, smaller superior/inferior accessory arteries, more than two RAs). In the case of multiple RAs, the vessel with the largest cross‐sectional area at the ostium was considered the main artery, and the rest as ‘accessory’; unless the difference between vessels' cross‐sectional area at the ostium was < 9 mm^2^, where they were then regarded as ‘equal’. Arteries < 1 mm in diameter were not accounted for. The terms ‘superior’ or ‘inferior’ accessory arteries describe its relationship to the origin of the main RA rather than the area of renal parenchyma which the vessel supplies. Any branching of the artery within 10 mm of the ostium is considered ‘early division’.

A small sample of patient images (*n* = 10) was analyzed by two additional independent assessors to assess the reliability of measurements.

### 2.4 Data Collection

All data were collected in an anonymised database on Microsoft Excel. In addition to data on vascular anatomy, basic demographic details (age, gender, ethnicity) were obtained from the Scottish Electronic Renal Patient Record. Details of whether or not each patient proceeded to donation (and, in those cases which did not, reasons for this) were also recorded.

### 2.5 Statistical Analysis

Data analysis was performed using IBM Statistical Package for Social Sciences (SPSS) version 28. Data were checked for normality and equal variance. Descriptive statistics were used to describe data with continuous variables summarized using mean (SEM). Categorical variables were summarized as proportions and frequencies. Frequency plots were used to highlight any outliers in arterial measurements such as angulation or maximum distance between renal arteries. Two‐sample *t*‐tests were used to compare arterial dimensions between the right and left kidneys. Chi Squared tests were performed to compare the proportion of kidneys with multiple vessels by laterality. Simple linear regression was used to test if age, gender, ethnicity, or number of arteries supplying the right kidney significantly predicted the number of arteries supplying the left kidney. The threshold for statistical significance was set at *p* < 0.05. Inter‐observer and intra‐observer agreement was assessed using a correlation coefficient.

## 3 Results

### 3.1 Population

**FIGURE 3:**
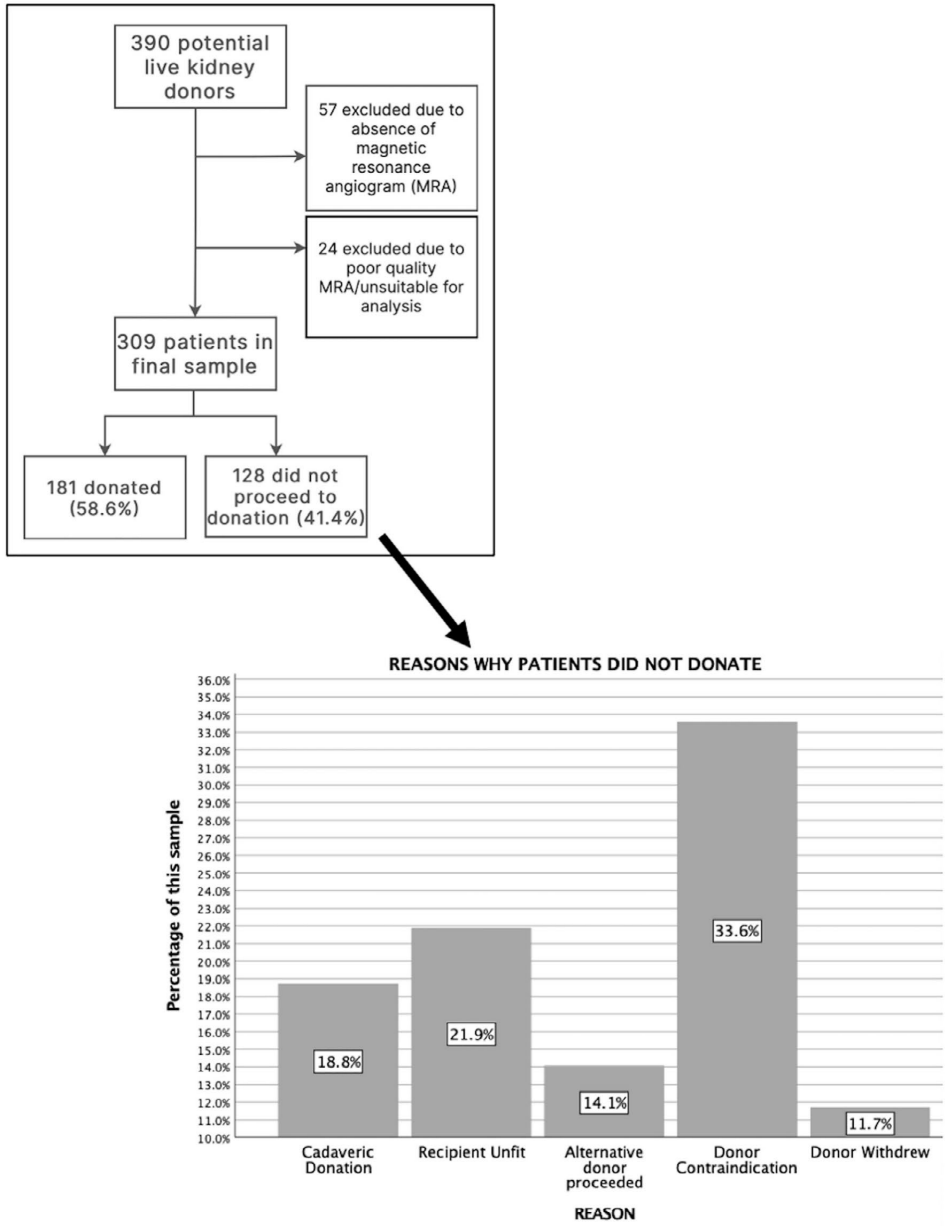
Study flow chart highlighting drop‐outs and exclusions. The table at the bottom right of the figure highlights reasons why patients who underwent work‐up imaging to be considered as a donor did not ultimately progress to donation. Relatively large numbers of drop‐outs highlight that this is a labour‐intensive process. Depicts the original sample size, exclusions, and the final number of potential donors included in the study. Additionally, the reason why certain patients from the final sample did not proceed with donation is outlined for interest.

Of the initial 390 potential living kidney donors, 81 individuals (20.77%) were excluded from analysis. Reasons for exclusion are outlined in Figure 3. A final sample size of 309 patients was obtained, allowing subsequent evaluation of 618 kidneys. Median age at donation was 50.8+/−12.0 years (range: 23–74). Males contributed 49.2% (*n* = 152) of this sample. 93.9% (*n* = 290) of patients were of white/European ethnicity. Of the initial sample, 58.6% (*n* = 181) proceeded to donation, with donor contraindications responsible for the majority of non‐proceeding cases (33.6%) (Figure 3).

### 3.2 Number of Renal Arteries

75.4% (*n* = 466) of kidneys were supplied by a single renal artery. This left 24.6% (*n* = 152) of the total sample possessing multiple RAs. 22.5% (*n* = 139) were supplied by two, and 2.1% (*n* = 13) by three RAs. There was no significant difference between left and right kidneys when comparing the proportion of kidneys supplied by a single RA (*p* = 0.959).

A total of 183 (59.2%) patients possessed single RAs bilaterally and 26 (8.4%) patients had multiple RAs bilaterally. 100 patients (32.4%) had a combination of single and multiple RAs. Of this sample, 40.8% of patients had multiple RAs supplying at least one kidney. Almost half (42.3%) of those with multiple RAs bilaterally did not proceed to donation.

### 3.3 Anatomical Configuration of Renal Arteries

**FIGURE 4:**
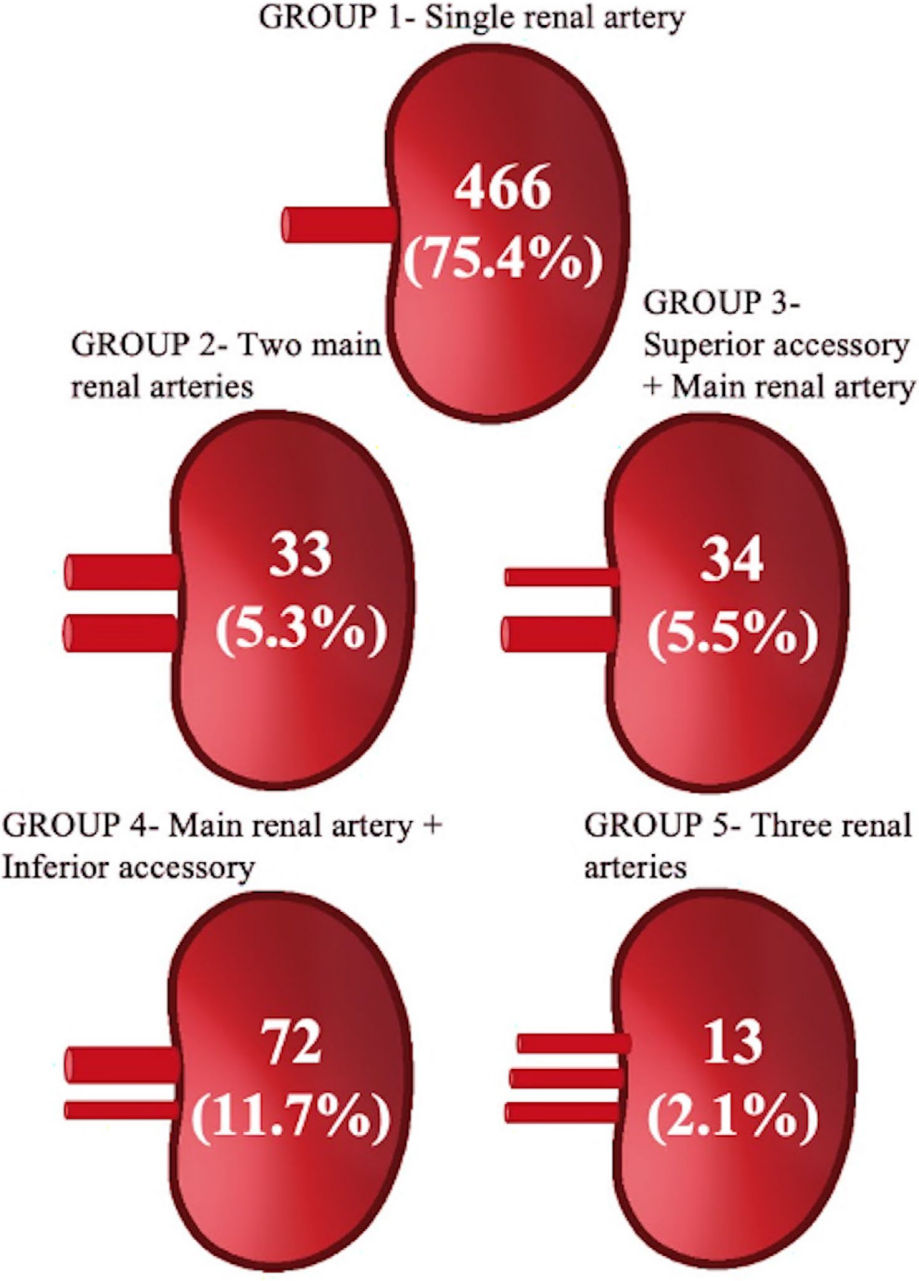
Anatomical groupings of kidneys based on the number and configuration of the renal arteries. The numbers represent the total counts and percentages of all kidneys falling into each group.

Kidneys were further divided into sub‐groups based on the anatomical configuration of the RAs (Figure 4). Mean cross‐sectional area, maximum and minimum diameters, length, angulation, and distance between each of the arteries (where applicable) of groups one to four are described in Table 1. These characteristics were compared between the left and right kidney (Table 2). Overall, main left RAs were larger (cross‐sectional area and maximum diameter) (*p* < 0.001–0.240), RAs of the right kidneys were longer (*p* < 0.001–0.018).

**TABLE 1:** Descriptive statistics of renal artery characteristics across groups one to four.

Group		Measurements	*N* (missing)	Mean	SEM	Range (max and min)	95% CI (mean)
One		Cross sectional area at ostium (mm^2^)	466 (0)	46.2355	0.47104	55.95 (19.06–75.01)	45.31, 47.16
		Maximum diameter (mm)	466 (0)	8.4623	0.04374	5.15 (6.23–11.38)	8.38, 8.55
		Minimum diameter (mm)	466 (0)	5.0643	0.03595	5.16 (3.18–3.84)	4.99, 5.13
		Length to first bifurcation (mm)	284 (182)	25.8915	0.68224	53.69 (2.29–55.98)	24.55, 27.23
		Length to hilum (mm)	466 (0)	42.2126	0.47507	60.74 (18.19–78.93)	41.28, 43.14
		Angle	466 (0)	125.15	0.585	79 (83–162)	124.00, 126.30
Two	Artery one	Cross sectional area at ostium (mm^2^)	33 (0)	33.1276	1.19097	31.17 (21.66–52.83)	30.70, 35.55
		Maximum diameter (mm)	33 (0)	7.2906	0.14891	4.50 (4.97–9.47)	6.99, 7.59
		Minimum diameter (mm)	33 (0)	4.5864	0.10159	2.22 (3.61–5.83)	4.38, 4.79
		Length to first bifurcation (mm)	18 (15)	25.5500	3.04677	49.29 (3.98–53.27)	19.12, 31.98
		Length to hilum (mm)	33 (0)	37.9455	1.78446	41.34 (20.67–62.01)	34.31, 41.58
		Angle	33 (0)	128.7879	2.52028	67.00 (93–160)	123.65, 133.92
	Artery two	Cross sectional area at ostium (mm^2^)	33 (0)	31.7906	1.28507	31.20 (20.05–51.25)	29.17, 34.41
		Maximum diameter (mm)	33 (0)	6.7388	0.21086	6.74 (2.30–9.04)	6.31, 7.17
		Minimum diameter (mm)	33 (0)	4.2009	0.11308	3.41 (2.00–5.41)	3.97, 4.43
		Length to first bifurcation (mm)	16 (17)	34.0488	3.25630	49.03 (7.51–56.54)	27.11, 40.99
		Length to hilum (mm)	33 (0)	45.4912	1.82582	39.05 (29.41–68.46)	41.77, 49.21
		Angle	33 (0)	112.82	2.569	65 (77–142)	107.58, 118.05
		Distance between arteries (mm)	33 (0)	20.3270	3.21481	78.40 (0–78.40)	13.78, 26.88
Three	Main	Cross sectional area at ostium (mm^2^)	34 (0)	44.4319	1.57454	34.24 (26.12–60.36)	41.23, 47.64
		Maximum diameter (mm)	34 (0)	8.3297	0.18081	3.90 (6.41–10.31)	7.96, 8.70
		Minimum diameter (mm)	34 (0)	4.9424	0.13399	3.09 (3.60–6.69)	4.70, 5.22
		Length to first bifurcation (mm)	20 (14)	28.2890	2.29420	37.29 (7.49–44.78)	23.49, 33.09
		Length to hilum (mm)	34 (0)	43.3456	1.96262	49.51 (23.81–73.32)	39.35, 47.34
		Angle	34 (0)	120.1765	2.30263	56.00 (81–137)	115.49, 124.86

	Accessory (superior)	Cross sectional area at ostium (mm^2^)	33 (1)	23.1321	1.30685	35.50 (10.60–46.10)	20.47, 25.79
		Maximum diameter (mm)	34 (0)	5.1279	0.25794	5.49 (2.60–8.09)	4.60, 5.65
		Minimum diameter (mm)	34 (0)	3.3559	0.17236	3.56 (1.40–4.96)	3.01, 3.71
		Length to first bifurcation (mm)	9 (25)	23.8333	3.57078	34.13 (9.55–43.68)	15.60, 32.10
		Length to hilum (mm)	34 (0)	41.7485	2.30599	65.74 (20.63–86.37)	37.06, 46.44
		Angle	34 (0)	120.4706	3.76238	101.00 (75–176)	112.82, 128.13
Distance between arteries (mm)		34 (0)	8.5588	1.27924	22.50 (0–22.50)	5.96, 11.16
Four	Main	Cross sectional area at ostium (mm^2^)	72 (0)	42.8399	0.92802	36.44 (29.65–66.09)	40.99, 44.69
		Maximum diameter (mm)	72 (0)	8.1128	0.09263	4.02 (6.69–10.71)	7.93, 8.30
		Minimum diameter (mm)	72 (0)	4.8542	0.07124	2.77 (3.89–6.66)	4.71, 5.00
		Length to first bifurcation (mm)	44 (28)	23.8957	1.50940	43.00 (5.11–48.11)	20.85, 26.94
		Length to hilum (mm)	72 (0)	41.0607	1.17947	43.68 (24.60–68.28)	38.71, 43.41
		Angle	72 (0)	121.53	1.250	47 (102–149)	119.04, 124.02
	Accessory (inferior)	Cross sectional area at ostium (mm^2^)	71 (1)	22.2356	0.76070	32.92 (9.71–42.63)	20.72, 23.75
		Maximum diameter (mm)	72 (0)	5.0806	0.16325	5.56 (2.20–7.76)	4.76, 5.41
		Minimum diameter (mm)	72 (0)	3.3842	0.10224	3.49 (1.5–4.99)	3.18, 3.59
		Length to first bifurcation (mm)	7 (65)	41.5500	2.67468	21.37 (34.30–55.67)	35.01, 48.09
		Length to hilum (mm)	72 (0)	49.7881	1.40178	55.05 (24.59–79.64)	46.99, 52.58
		Angle	70 (2)	101.64	2.599	85 (45–130)	96.46, 106.83
		Distance between arteries (mm)	72 (0)	26.4863	3.09816	114.00 (0–114)	19.39, 30.91

*Note:* Analysis includes parameters such as cross‐sectional area, maximum and minimum diameters, length to first bifurcation, length to hilum, and angulation.

**TABLE 2:** Comparison of renal artery characteristics between left and right kidneys across groups one to four.

Group		Measurements	Left	Right	*p*
			Mean (SEM)	Mean (SEM)	
One		Cross sectional area at ostium (mm^2^)	48.3044 (0.66902)	44.1667 (0.63636)	**< 0.001**
		Maximum diameter (mm)	8.6021 (0.06098)	8.3226 (0.06151)	**0.001**
		Minimum diameter (mm)	5.1315 (0.05056)	4.9970 (0.05084)	0.061
		Length to first bifurcation (mm)	23.1157 (0.83458)	28.3364 (1.01324)	**< 0.001**
		Length to hilum (mm)	37.4858 (0.57832)	46.9394 (0.61454)	**< 0.001**
		Angle	120.90 (0.707)	129.39 (0.847)	**< 0.001**
Two	Artery one	Cross sectional area at ostium (mm^2^)	35.4426 (1.61148)	29.9857 (1.42524)	**0.010**
		Maximum diameter (mm)	7.4426 (0.23133)	7.0843 (0.14970)	0.240
		Minimum diameter (mm)	4.6647 (0.11986)	4.4800 (0.17714)	0.377
		Length to first bifurcation (mm)	20. 9388 (2.96526)	29.2390 (4.76322)	0.183
		Length to hilum (mm)	32.7063 (1.64658)	45.0557 (2.57427)	**< 0.001**
		Angle	126.9474 (2.82685)	131.2857 (4.58309)	0.403
	Artery two	Cross sectional area at ostium (mm^2^)	34.5247 (1.52402)	28.0800 (1.83787)	**0.011**
		Maximum diameter (mm)	7.1658 (0.18026)	6.1593 (0.38994)	**0.016**
		Minimum diameter (mm)	4.3474 (0.12009)	4.0021 (0.20448)	0.133
		Length to first bifurcation (mm)	34.0130 (4.51506)	34.1083 (4.86516)	0.989
		Length to hilum (mm)	41.7474 (2.09286)	50.5721 (2.76814)	**0.014**
		Angle	113.74 (2.618)	111.57 (5.022)	0.684
		Distance between arteries (mm)	24.2321 (4.59987)	15.0271 (4.05624)	0.160
Three	Main	Cross sectional area at ostium (mm^2^)	47.0650 (1.85667)	41.4694 (2.46778)	0.076
		Maximum diameter (mm)	8.5489 (0.22501)	8.0831 (0.28396)	0.203
		Minimum diameter (mm)	5.2411 (0.18997)	4.6062 (0.15392)	**0.016**
		Length to first bifurcation (mm)	25.4267 (2.80138)	32.5825 (3.58166)	0.130
		Length to hilum (mm)	39.0411 (2.36545)	48.1881 (2.81434)	**0.018**
		Angle	124.111 (2.00797)	115.7500 (4.14478	0.083
	Accessory (superior)	Cross sectional area at ostium (mm^2^)	24.8041 (2.11605)	21.3556 (1.42530)	0.192
		Maximum diameter (mm)	4.9800 (0.39880)	5.2944 (0.32371)	0.551
		Minimum diameter (mm)	3.1628 (0.25858)	3.5731 (0.21838)	0.240
		Length to first bifurcation (mm)	25.5175 (6.27248)	22.4860 (4.61238)	0.702
		Length to hilum (mm)	36.4617 (2.78978)	47.6963 (3.23758)	**0.013**
		Angle	113.1111 (3.72142)	128.7500 (6.31566)	**0.036**
		Distance between arteries (mm)	8.0206 (1.93685)	9.1644 (1.67967)	0.331
Four	Main	Cross sectional area at ostium (mm^2^)	45.8312 (1.47589)	40.3087 (1.02504)	**0.002**
		Maximum diameter (mm)	8.2818 (0.14368)	7.9697 (0.11709)	0.093
		Minimum diameter (mm)	4.9267 (0.10085)	4.7928 (0.10022)	0.353
		Length to first bifurcation (mm)	17.9833 (1.41359)	29.2939 (2.02111)	**< 0.001**
		Length to hilum (mm)	33.9276 (1.19796)	47.0964 (1.29782)	**< 0.001**
		Angle	120.73 (1.394)	122.21 (1.994)	0.273

	Accessory (inferior)	Cross sectional area at ostium (mm^2^)	22.2267 (1.29426)	22.2434 (0.88735)	0.991
		Maximum diameter (mm)	5.0409 (0.26823)	5.1141 (0.20149)	0.825
		Minimum diameter (mm)	3.3727 (0.17097)	3.3938 (0.12339)	0.919
		Length to first bifurcation (mm)	37.7500 (0.8000)	43.0700 (3.59688)	0.418
		Length to hilum (mm)	44.4603 (1.55857)	54.2962 (1.96983)	**< 0.001**
		Angle	105.34 (4.226)	98.53 (3.167)	0.193
Distance between arteries (mm)		25.6955 (4.20962)	27.1554 (28.24551)	0.816

*Note:* This table illustrates a detailed comparison of the anatomical characteristics of the RAs from the left and right kidneys, demonstrating the longer right RA with greater distance to first bifurcation but slightly wider (larger) diameter of a single left RA. Bold values are those deemed statistically significant (*p* < 0.05).

### 3.4 Group One

Over three‐quarters of kidneys (75.4%) had a single RA. This was evenly distributed between the left and right sides (*n* = 233 left, *n* = 233 right). Thirty‐three kidneys (11.6%) in group one possessed a single RA that branched early, defined as bifurcation within 10 mm of the ostium.

### 3.5 Group Two

Of the sample, 5.3% (*n* = 33) (19 left and 14 right kidneys) had dual RAs of similar caliber, hence fell into group 2. Mean cross‐sectional area was 33.13 mm^2^ (±1.19 mm^2^) for artery one and 31.79 mm^2^ (±1.29 mm^2^) for artery two. This is smaller than the mean cross‐sectional area for other groups where there was only one dominant RA. Within this group, there tended to be a longer distance to the first bifurcation in right kidneys (*p* < 0.001) (Table 2).

### 3.6 Group Three

Group three comprised a total of 5.5% (*n* = 34) of kidneys, with one small superior accessory and a lower main RA found in 18 left and 16 right kidneys. The mean cross‐sectional area of the main artery in this group was 44.43 ± 1.57 mm^2^. The superior accessory artery in this group had the smallest mean minimum diameter of all RAs, measuring 3.36 mm (±0.17 mm).

### 3.7 Group Four

Group four included 11.7% (*n* = 72) of kidneys; each with one upper main and one lower accessory artery. The distribution included 33 left kidneys and 39 right kidneys. The mean cross‐sectional area of the main artery in this group was 42.84 ± 0.93 mm^2^. The inferior accessory artery in this group on average had the longest course, showing the greatest mean distance to the hilum of all arteries (49.79 ± 1.40 mm). One lower polar artery originated from the common iliac artery. All others originated from the abdominal aorta.

### 3.8 Group Five

A total of 13 kidneys (2.1%) possessed three RAs, comprising six left kidneys and seven right kidneys. The main RA was the superior‐most artery (artery one) in five kidneys and the middle artery (artery two) in eight kidneys. The inferior artery (artery three) was never identified as the main artery. Of the seven arteries (17.9%) which bifurcated prior to the hilum, none showed early branching (< 10 mm), with a range of 17.0–38.0 mm. The distance between the origins of artery one and two ranged from 0 to 35.1 mm, and 0 to 78.9 mm between artery two and three. The minimum and maximum total distances between arteries one and three were 8.4 and 85.2 mm, respectively. Angulation for the main artery ranged from 82° to 143°.

### 3.9 Accessory Arteries

There was no statistically significant difference in the incidence of superior accessory arteries (group three) (*p* = 0.724) and inferior accessory arteries (group four) (*p* = 0.452) between the right and left kidneys.

### 3.10 Distance Between the Origin of Renal Arteries

In kidneys with two RAs (groups two, three, four), the mean distance between the origins of these vessels was 19.9 ± 1.8 mm (range: 0–114 mm). In the kidney where arterial origins were 114 mm apart, the inferior accessory artery originated from the common iliac artery.

**FIGURE 5:**
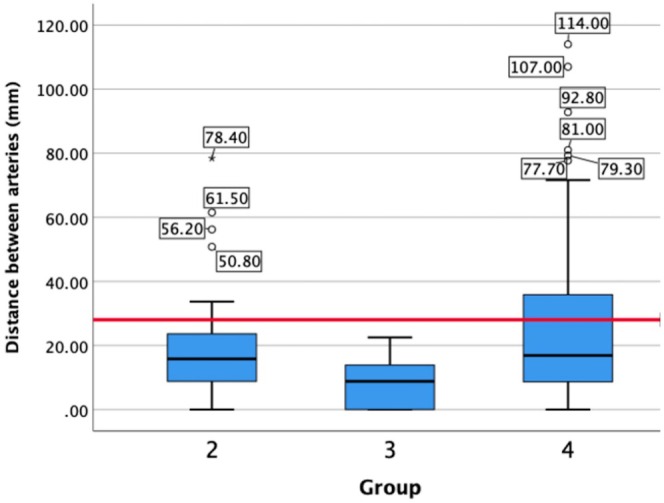
Distances between the origin from the aorta in both renal arteries in each kidney in groups two, three, and four. Outliers are also displayed with their values noted (mm). The red reference line at 28 mm indicates the length of the current clamp used for NMP (Walters clamp), with all values above this line unable to be perfused on this single clamp.

The standard clamp used for deceased donor transplantation and NMP is 28 mm in length. Figure 5 displays the distance between arterial origins in kidneys with two RAs (groups two, three, and four) with the reference line at 28 mm signifying the cut‐off for compatibility with the current clamp. Of the 605 kidneys with either one or two arteries, 33 kidneys (5.5%) had two RAs greater than 28 mm apart. Fourteen kidneys (2.3%) had distances of ≥ 28 to ≤ 40 mm between arterial origins, and nine kidneys (1.5%) had distances 40–60 mm. The remaining 10 kidneys (1.7%) had distances ≥ 60 mm between arterial origins. All of the kidneys in group three, i.e., small superior accessory RA, and 30.8% of kidneys with three vessels had RAs ≤ 28 mm apart. Overall, 94.5% of kidneys with one or two RAs in this sample had vessels compatible with the standard clamp. Of those with two RAs, 23.74% had distances greater than 28 mm.

### 3.11 Angulation From Aortic Origin

**FIGURE 6:**
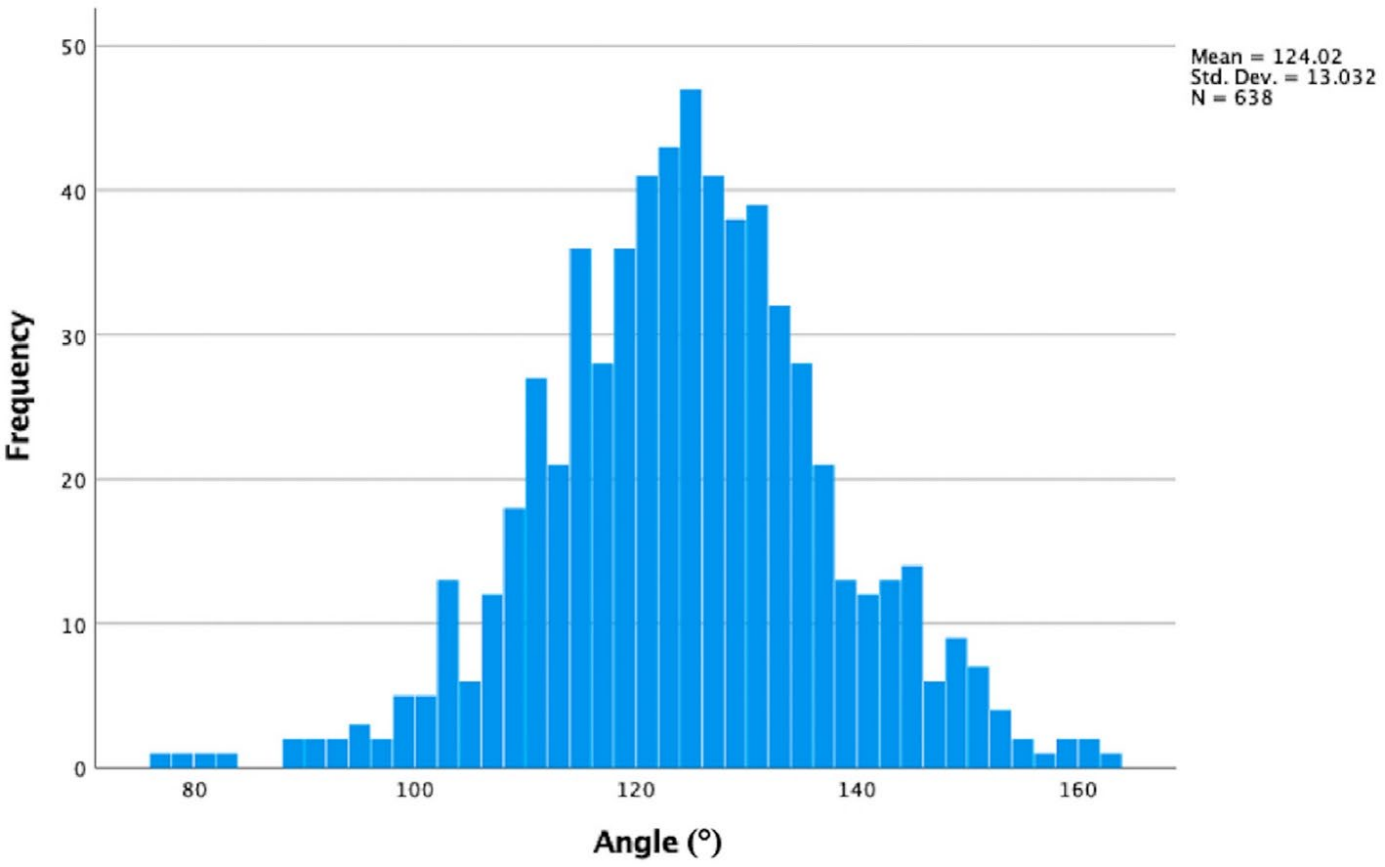
Histogram outlining the distribution of the angulation (°) of renal arterial origins.

Only six kidneys (0.9%) with one or two RAs had vessels that left the aorta at an acute angle (≤ 90°). The remaining 632 kidneys had a main RA that originated at an angle greater than 90°. The most acute angle identified was 77° (group two), and the most obtuse was 162° (group one). The complete distribution of these values can be seen in Figure 6. Among the main arteries, 122 (19.12%) exhibited angulations of ≥ 135°, including 19 (3.0%) with angles of 150° or greater.

### 3.12 Comparison Between Left and Right Kidneys

The number of arteries supplying the left kidney could be predicted by the number of arteries supplying the right kidney (*β* = 0.15), but this model was of poor fit. The overall regression was statistically significant (*R* ^2^ = 0.24, *F* (1,307) = 7.41, *p* = 0.007). The fitted regression model was: Number of left arteries = 1.017 + (number of right arteries) × 0.150.

There was no statistically significant correlation between the number of arteries on the left kidney and either age, sex, or ethnicity (*p* = 0.32, *p* = 0.46 and *p* = 0.14, respectively).

### 3.13 Inter and Intra‐Observer Reliability

Both inter‐and intra‐observer agreement was strong, with kappa coefficients ranging from 0.95 to 0.99 and 0.91 to 0.99, respectively. These results were statistically significant (*p* < 0.001).

## 4 Discussion

In this study, we evaluated the renal arterial anatomy of potential living kidney donors, as visualized on CE‐MRA. This patient cohort served as a surrogate for the wider Scottish kidney donor population (both living and deceased). We have described the observed anatomy in this contemporaneous patient cohort and made inferences about how this might impact the application and delivery of NMP.

### 4.1 Key Findings

Nearly a quarter of kidneys (24.4%) had multiple RAs. In most centres, this ‘complex anatomy’ would be considered a relative contraindication to NMP at present. Even in patients with single RAs the presence of early branching was identified in more than 10%, potentially introducing further surgical complexities. Almost a third of kidneys with multiple arteries had too large a distance between RAs (≥ 28 mm) to be compatible with the current clamp for perfusion. Left renal arteries tended to be large, whilst right renal arteries were longer. Furthermore, a longer length of common arterial stem before initial bifurcation was evident in right kidneys. Many vessels had an obtuse angulation of their aortic origin, with implications for cannula design if individual vessels were to require cannulation.

Pre‐operative imaging of living kidney donors was assumed to be representative of the wider Scottish deceased donor population, the kidneys from whom would likely be the ones considered for NMP. Despite this study including only healthy individuals, the results are assumed to be representative and generalizable to the wider adult population in the West of Scotland since renal arterial anatomical variations will not differ with age or disease.

### 4.2 Existing Evidence Base and Context

**FIGURE 7:**
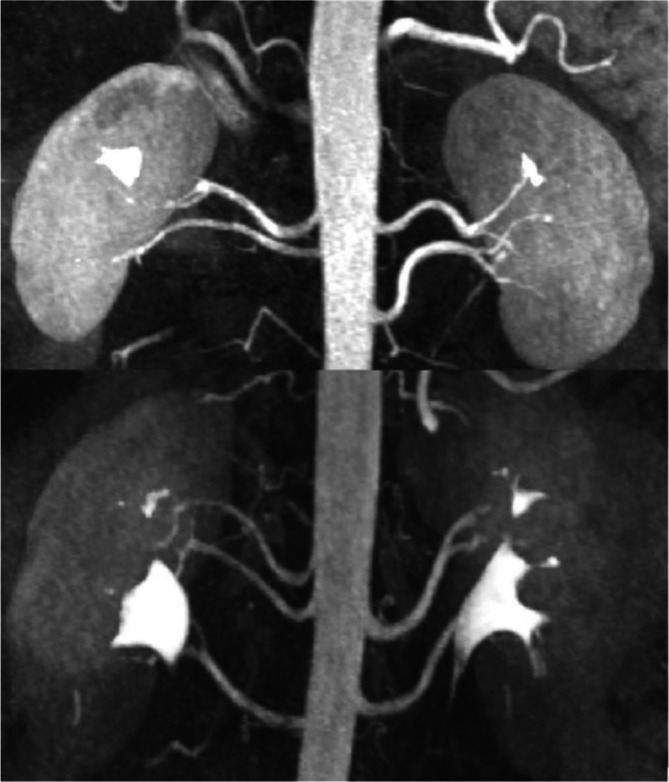
Example CE‐MRA images taken from PACS showing two pairs of kidneys display multiple renal arteries bilaterally. The superior pair of kidneys possesses two renal arteries of similar caliber on both the right and the left side (i.e., Group two). The inferior pair of kidneys has two renal arteries supplying the left kidney (Group two), and three supplying the right kidney (Group five).

Over 40% of patients in this sample had at least one kidney supplied by multiple RAs. These findings are in keeping with previous studies (Pradhay et al. 2021; Sampaio and Passos 1992; Johnson et al. 2013; Mihaylova et al. 2023). Mihaylova et al. observed that accessory RAs were present in 41.2% (*n* = 231) of subjects (Mihaylova et al. 2023). Similarly, our study observed that 8.4% of patients had multiple renal arteries bilaterally (example CE‐MRA images demonstrating multiple RAs in Figure 7). Similar rates of bilateral ‘complex’ anatomy were observed by both Mihaylova et al. (8.6%) and Mir et al. (10.2%) (Mihaylova et al. 2023; Mir et al. 2008). Nonetheless, significant heterogeneity exists in the overall frequency of multiple RAs reported, with rates as low as 4% demonstrated in systematic reviews by both (Natsis et al. 2014; Gulas et al. 2018). Differences in imaging modalities (e.g., radiological, surgical, cadaveric), patient populations and classifications/nomenclature may explain some of the variation in prevalence rates reported. Although the maximum number of RAs noted in this study was three (one main and two accessories), previous literature has reported more (Johnson et al. 2013; Mihaylova et al. 2023; Natsis et al. 2014; Gulas et al. 2018). The presence of multiple RAs poses increased risk at time of transplantation (prolonged anastomotic time, more warm ischaemia and increased risk of early graft thrombosis) as well as technical challenges for NMP.

### 4.3 Significance of the Lower Polar Artery

In this sample, more than twice the number of kidneys with multiple RAs were supplied by an additional RA originating inferiorly to the main (Group four) than superiorly (Group three). This finding is consistent with previous studies where inferior accessory arteries were the most common variant encountered (Johnson et al. 2013; Guan et al. 2013; Bordei et al. 2004). The inferior‐originating arteries in this study have a high likelihood of supplying the lower pole of the renal parenchyma, hence contribute to the ureteric blood supply. This is surgically relevant because particular care must be taken with these arteries to maintain integrity and avoid damage. Disruption to this blood supply can cause ischaemic complications to the ureter leading to ureteric ischaemia and subsequent stricture and/or urine leak post‐transplant (Johnson et al. 2013; El‐Sherbiny et al. 2008; Sebastià et al. 2010). The decision to classify accessory RAs based on their origin rather than polar supply relates to the purpose of this study in exploring how to improve NMP equipment. It should be noted, however, that simply because an accessory RA arises inferiorly from the abdominal aorta, it does not necessarily supply the lower pole of the kidney, nor the ureter.

### 4.4 Potential Limitations

Radiological investigations are paramount in the work‐up pathway for potential living kidney donors. They allow the surgical team to understand individuals' renal vascular anatomy and identify variations such as multiple RAs or early branches and plan both donor and recipient surgery accordingly. The accuracy of CE‐MRA in arterial evaluation has been demonstrated, correlating well with other imaging modalities and intra‐operative findings. However, CE‐MRA may be less accurate for accessory arteries of small caliber (< 1 mm) (Monroy‐Cuadros et al. 2008; Engelken et al. 2013; Asgari et al. 2011). RAs < 1 mm were excluded from this study based on their unlikely provision of substantial blood supply to the kidney. They were considered to be surgically irrelevant, with most, if not all, tied off during surgery rather than implantation attempted. CE‐MRA accuracy is influenced by factors such as respiratory movement or poor contrast timing, reducing the quality of branch vessel projection and analysis potential (Monroy‐Cuadros et al. 2008; Engelken et al. 2013; Asgari et al. 2011). 6.2% (*n* = 24) of the initial sample were excluded due to inadequate images. Studies of this nature, involving radiographic interpretations, depend on a level of subjective interpretation by observers. To counter this, all images in this study were assessed by two independent observers with strong inter/intra‐observer agreement, reducing the risk of measurement bias and supporting the precision and reliability of the results collected. Furthermore, the decision to analyze only CE‐MRAs (and not other imaging modalities such as CTA) reduced variation which may exist between imaging modalities. This did, however, result in the exclusion of 14.6% of patients, reducing the sample size moderately. Future studies may explore the possibility of designing robust methods that allow analysis of both imaging modalities simultaneously.

Measuring the angulation of the RA as it leaves the aorta in the coronal image necessitates viewing a three‐dimensional variable in two dimensions. This may not be truly representative of the structure and may restrict the ability to make surgically meaningful inferences on this characteristic. Furthermore, measurements between vessels leaving the aorta were made on the coronal view of the image. This assumes vessels are directly vertically aligned, when in reality often one vessel originates more anteriorly/posteriorly than the other. This may lead to potential underestimation of the true distance between vessels.

The majority of this sample were of white/European ethnicity. It is unclear if these findings are representative of wider populations. There remains a possibility that renal vascular variations may be more prevalent in certain groups of people (Johnson et al. 2013; Natsis et al. 2014; Gulas et al. 2018). However, in this study, linear regression did not show a significant relationship between ethnicity and presence of multiple arteries.

### 4.5 Implication of Findings to NMP

**FIGURE 8:**
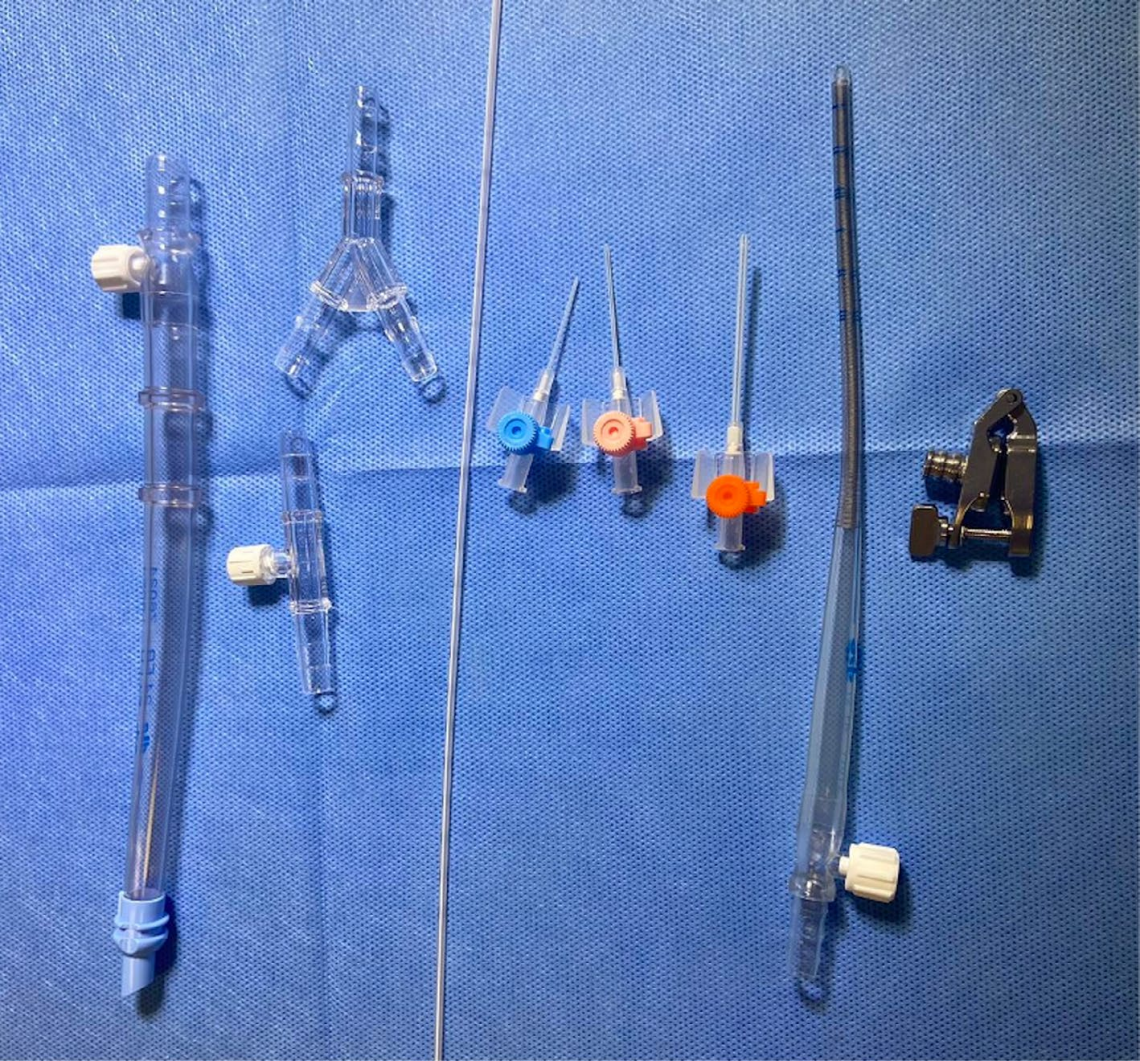
Existing range of cannulae and clamps utilized in practice at our centre for renal artery cannulation on the NMP circuit. From left to right: Repurposed oxygen tubing; straight and Y‐connectors (the Y‐connector is occasionally used to join multiple pieces of tubing if there is a kidney with multiple vessels requiring perfused), pediatric umbilical catheter; intravenous cannulae in a range of sizes (24F (blue), 20F (pink), 16F (orange); repurposed chest drain; Walters clamp. It is evident that much of this equipment has been repurposed from other areas of practice and is often suboptimal for NMP. For example, current devices offer little flexibility or adaptability to overcome variations in RA anatomy.

As previously discussed, NMP has ground‐breaking potential for both reconditioning and assessment of marginal kidneys prior to transplantation. By increasing confidence in organ quality and with the potential to resuscitate or recondition a suboptimal organ, it may ultimately prove valuable in increasing the numbers of kidneys available for transplantation. Current equipment used for perfusion on the NMP circuit (Figure 8) is often repurposed from other areas of clinical practice, with nothing being the perfect fit or uniquely designed for the purpose of NMP specifically. Many of the current options prove suboptimal for multiple arteries. For example, currently the standard Walter's clamp used at our centre cannot facilitate multiple arteries that originate more than 28 mm apart. This would exclude nearly one in three kidneys with multiple vessels. If, however, the clamp was 40 mm long, 14 more kidneys (2.3%) from this sample could be perfused, whilst increasing to 60 mm would permit a further nine kidneys. In cases where vessels are situated at a distance unsuitable for a single clamp, multiple clamps may be required. This may be particularly true when there are greater than two RAs present. Over a third of kidneys with three arteries had a cumulative distance between artery one and three of ≥ 28 mm. Additionally, the harsh metal clamp can cause trauma to surrounding tissues. Damaged tissue must be removed, reducing the surgical availability of tissue for an anastomotic patch at implantation. Finally, RAs that leave the aorta at very acute or obtuse angulations are often difficult to cannulate due to the rigidity of current cannulae. The results from this sample demonstrate that a wide range of angulations (45°–176° in this sample) can be encountered when cannulating renal arteries, with a large number of arteries originating at obtuse angles. Cannulae that are softer and more flexible are required to avoid arterial trauma in RAs with non‐conventional angulations.

## 5 Conclusion

This study demonstrates relevant clinical anatomy of the RAs and highlights its application to the development of tools and devices to facilitate NMP. Specifically, the following points have been highlighted:

1. Almost a quarter of kidneys have multiple RAs and, as such, pose technical challenges for NMP.
2. Over 20% of kidneys with multiple RAs have aortic RA origins further apart than can be cannulated with the existing clamps commercially available.
3. There is a wide variation in the angulation of RAs as they originate from the aorta, with many arteries having obtuse angulation.
4. In patients with two similarly sized RAs, right kidneys have a longer length of common stem before the first bifurcation, potentially making them easier to effectively cannulate.

These observations highlight the need for the development of new devices for RA cannulation on NMP. In particular, clamps serving a longer length of vessel and soft, malleable cannulae capable of bending to accommodate angulation without kinking would be of particular benefit. Development of such technology is necessary if NMP is to be available for all kidneys, not just ones with simple anatomy. This anatomical study informs the development of such devices.

## Disclosure

Appropriate approvals have been sought and obtained. Statements to this effect are contained within individual figure legends.

## Ethics Statement

The Health Research Authority (HRA) online tool determined that this retrospective study using anonymised patient data did not require formal approval from the Research Ethics Committee (REC). Caldicott guardian approval was obtained for data sharing.

## Consent

The authors have nothing to report.

## Data Availability

The data supporting the findings reported in this paper are available under a CC0 licence from the BioStudies repository at https://doi.org/10.6019/S‐BSST2006.

## Appendix A Standard Operating Procedure for Taking Measurements on VUE PACS 3D Vessel Analysis Package

**Table:**

Enter patient archive explorer. Select “MRA Renal and Visceral”
2 From the patient mini archive—select “Angio3D_post_arterial”
3 Double click the image to make it full screen, then change view to MipPR by pressing the **“I”** key
4 In the coronal plane, count the number of arteries entering the hilum of each kidney
5 Right click “View As”‐“Vessel Analysis”
6 Right click on the top left image—“change image” → “Angiography MIP”
7 Click on “Vessel” on the top bar, then select the “Define New Vessel” icon on ribbon in the “definition” category → “General Vessel Protocol”
8 Using axial MPR, begin higher than origin of any renal arteries by scrolling through the image, place cross in centre of aorta and descend placing crosses at regular intervals until below level of potential renal arteries
9 Select “Define New Vessel” on ribbon—“General Vessel Protocol” again. This time locate where the most superior (if multiple) renal artery leaves the aorta in the axial plane and place your first point by left clicking in the midline of the renal artery just at the ostium. Scroll through the image slowly following the course of the artery, placing points (by left clicking) at regular intervals in the midline of the artery until it enters the renal hilum. To complete the vessel, either double click as the last point is placed or alternatively select the green tick in the new vessel pop‐up box (bottom left corner of the screen).
10 Using newly generated cross‐sectional image of vessel, the software automatically generates measurements for estimated cross sectional area at the ostium (mm^2^), maximum and minimum diameter (mm) at any point along the vessel before any bifurcations. (These are usually displayed in the bottom right image of the screen)
11 Use the vessel analysis panoramic image (bottom left) to determine location of earliest bifurcation by moving the vessel (left click and hold over the image and move the mouse). Once bifurcation has been identified, slide through the vessel using the scroller on the mouse until the small line on the centre line of the vessel is located at the ostium (refer to all other images on the screen to check this). Then select the “curve measurement” tool from the “graphics” ribbon. Place first point by clicking near the ostium, using the line as reference. (Ensure the correct curved line tool icon is shown at the cursor before clicking). Then work distally, placing points along the centre line of the vessel, stopping just prior to the vessel bifurcation by again double clicking. Note the length of this line. If no bifurcation occurs prior to the hilum, note this as “n/a”.
12 Next, move back to initial Coronal MipPR image by selecting “Viewer” in the top left corner of the screen (above the main ribbon).
13 Place the cross at the origin of the relevant renal artery, use the same swivel tool as previously mentioned (bottom left of screen), and left click and hold over it while moving the mouse to manipulate the image until the entire length of renal artery can be seen from the aorta to the kidney. For consistency, the hilum is defined using a line running in the maximum long section of the kidney parenchyma, so use the “straight line” tool from “graphics” to depict this. Using this line as a guide, measure the length along the midline of the renal artery from the ostium to the hilum using the curved measurement tool as done in step 11.
14 Next, right click over the swivel icon in the bottom left and select “original” to return to the original coronal view. With the cross placed on the renal ostium again, use the swivel tool to rotate the image until both upper and lower sections of the aorta and bifurcation are clearly shown, and the renal arteries can be seen exiting the aorta on either side. Then select the “angle tool” from graphics. Left click to place the first point in the centre of the renal artery about 5 mm along, then place the second point in the centre of the aorta, following the angle line of the artery. Finally, place the 3rd point superiorly in the midline of the aorta, ensuring the line follows the direction and midline of aorta. Note the angle here as the renal artery leaves the aorta.
15 This final step is only relevant if there are multiple arteries unilaterally. Returning to the coronal “viewer” as in step 12, use the straight‐line tool to measure the distance between the midline of each artery. Also note the closest distance the arteries come together in their course using the same straight‐line tool in the coronal view.
16 If there are multiple vessels on the left side, repeat steps 9–15.

*Note:* Once all vessels on the left side are analyzed, move on to repeat the same steps 9–16 on all arteries on the left side.
